# Comparative Analysis of Clinical and Radiologic Staging of Cervical Cancer: A Cross-Sectional Study in Ethiopia

**DOI:** 10.4314/ejhs.v34i1.4S

**Published:** 2024-10

**Authors:** Fami Zekeriya Yusuf, Tesfaye Kebede, Michael Teklehaimanot Abera, Alemayehu Bedane, Assefa Getachew, Semira Abrar

**Affiliations:** 1 Adama Comprehensive Specialized Hospital Medical College, Department of Radiology, Adama, Ethiopia; 2 Department of Radiology, School of Medicine, Addis Ababa University, Addis Ababa, Ethiopia; 3 Department of Radiology and Medical Radiologic Technology, School of Medicine, St. Paul's Hospital Millennium Medical College, Addis Ababa, Ethiopia

**Keywords:** Advanced cervical cancer, cervical cancer, computed tomography, FIGO, magnetic resonance imaging, Tikur Anbessa Specialized Hospital (TASH)

## Abstract

**Background:**

Cervical cancer remains a significant challenge in developing countries, with many patients diagnosed at advanced stages. The clinical staging of cervical cancer is guided by the International Federation of Obstetrics and Gynecology (FIGO) guidelines, while computed tomography (CT) and magnetic resonance imaging (MRI) offer valuable supplemental information. This study aimed to evaluate the initial clinical and imaging stages of cervical cancer and to assess the agreement between these staging methods.

**Methods:**

A cross-sectional study was conducted involving 115 newly diagnosed cervical cancer patients at Tikur-Anbessa Specialized Hospital from September 1, 2022, to February 30, 2023. Clinical staging was performed for all patients, with CT staging for 107 and MRI staging for 34. Data were extracted from the hospital's central databases and analyzed using SPSS version 27. Descriptive and reliability analyses were conducted, with statistical significance set at a p-value of <0.05 and a 95% confidence interval.

**Results:**

At diagnosis, 61 patients (53%) presented with advanced clinical stages (III-IV). CT imaging indicated advanced stages in 85 patients (73.9%), while MRI was performed on 34 patients. Agreement between clinical and CT-based staging was poor (weighted Cohen's kappa = 0.171, p = 0.016), while agreement between clinical and MRI-based staging was moderate (weighted Cohen's kappa = 0.418, p = 0.007).

**Conclusion:**

Newly diagnosed cervical cancer patients exhibit a high prevalence of advanced-stage disease. There is poor agreement between clinical and CT-based cervical cancer staging, contrasted with moderate agreement between clinical and MRI-based staging.

## Introduction

Cervical cancer is a largely preventable and treatable malignancy, predominantly caused by persistent infection with high-risk strains of human papillomavirus (HPV) (1, 2). It ranks as the fourth most common cancer among women globally, with the highest rates occurring in developing countries (3). The long latency period of cervical cancer makes it amenable to virological and cytological screening, which has successfully reduced incidence rates in many developed nations (4).

In many developing countries, cervical cancer is the most prevalent cancer among middle-aged women and a leading cause of cancer-related deaths. This is largely attributed to limited access to healthcare and insufficient routine screening (5, 6). In Ethiopia, only 14.79% of eligible women have undergone cervical cancer screening (7), contributing to cervical cancer being the second-most common and deadly cancer among women in the country (2). According to Begoihn et al. (8), out of 6,300 new cases diagnosed annually, 4,884 result in death. Delayed diagnosis, often linked to poor awareness and rural residency, poses a significant challenge (9).

Clinical staging adheres to FIGO guidelines (Table 1), which are crucial for determining management strategies and prognoses (10, 11). However, clinical evaluation often inadequately assesses parameters such as parametrial invasion, tumor size, lymph node involvement, and distant metastasis (12, 13). Inadequate staging occurs in 20-40% of cases (16). Modern imaging techniques, including CT and MRI, are recommended to address these gaps (12, 14). CT offers an overall accuracy of 65% and is particularly effective in detecting metastatic lymph nodes (15). It is also valuable for diagnosing hydronephrosis, indicative of stage III disease (16). However, previous studies have highlighted the limitations of CT, such as difficulty in visualizing certain cervical tumors due to their similar density to surrounding stroma (17). In contrast, MRI demonstrates greater sensitivity in detecting parametrial and lymph node involvement due to its superior soft-tissue resolution (18).

**Table 1:** FIGO staging for cervical cancer. Based on Bhatla N, Aoki D, Sharma DN, Sankaranarayanan R.: Cancer of the cervix uteri: 2021 update. Int J Gynaecol Obstet 155 Suppl 1:28-44, 2021 (19)

Stage	Description
I	Tumor confined to cervix (extension to uterine body should be disregarded)
IA	Invasive tumor that can be diagnosed only by microscopy, maximum depth of stromal invasion < 5 mm
IA1	Maximum depth of stromal invasion is < 3 mm
IA2	Maximum depth of stromal invasion is ≥ 3 and < 5 mm
IB	
IB1	Invasive tumor ≥ 5 mm and < 2 cm in greatest dimension
IB2	Invasive tumor ≥ 2 cm and < 4 cm in greatest dimension
IB3	Invasive tumor ≥ 4 cm in greatest dimension
II	Tumor extends beyond the uterus (no involvement of lower third of vagina or pelvic sidewall)
IIA	Involvement up to upper two-thirds of vagina, no parametrial involvement
IIA1	Invasive tumor < 4 cm
IIA2	Invasive tumor ≥ 4 cm
IIB	Parametrial involvement not to the pelvic wall
III	Involvement of lower third of vagina, extension to the pelvic wall, hydronephrosis or nonfunctioning kidney, lymph node involvement (irrespective of tumor size and extent), or some combination of these
IIIA	Involvement of lower third of vagina but no pelvic wall involvement
IIIB	Extension to pelvic wall, hydronephrosis or nonfunctioning kidney (not secondary to other causes), or both
IIIC	Pelvic and paraaortic lymph node metastasis (irrespective of tumor size and local extension)
IIIC1	Pelvic lymph node metastasis
IIIC2	Paraaortic lymph node metastasis
IV	Extension beyond the true pelvis or biopsy-proven involvement of bladder or rectal mucosa
IVA	Involvement of adjacent pelvic organs
IVB	Distant metastasis

Note—When the appropriate stage is in doubt, the lower stage should be assigned. In respect to tumor size and extent, when available, imaging and pathologic findings can be used to supplement clinical findings, with an added notation of r (imaging) and p (pathology). FIGO = International Federation of Gynecology and Obstetrics. Adapted with permission from [6].

This study aims to assess the initial staging of cervical cancer at diagnosis, identify discrepancies between clinical and imaging methods, and evaluate the agreement between these approaches.

## Materials and Methods

Study Design, Period, and Area: A cross-sectional study was conducted at Tikur Anbessa Specialized Hospital (TASH) in Addis Ababa, Ethiopia, from September 1, 2022, to February 30, 2023. TASH is the largest cancer referral center in the country, managing approximately 80,000 new cases annually and housing one of the few radiotherapy facilities.

**Study population and sampling method**: The study included all cervical cancer patients who visited TASH during the study period and met the inclusion criteria. A convenience sampling method was employed, resulting in a final sample size of 115 patients. Selection bias may be present due to the over-representation of patients with easier access to TASH.

Patients received initial clinical staging based on FIGO guidelines, followed by imaging studies. Radiologists provided their assessments after the clinical stage and histopathology results were available.

**Inclusion and exclusion criteria**: Eligible patients included those with a pathologically confirmed cervical cancer diagnosis for the first time within the study period who completed clinical and imaging evaluations. Patients with incomplete records, those with known cervical cancer undergoing follow-up, and those with concurrent malignancies receiving chemotherapy or radiotherapy were excluded. This exclusion may underestimate the true prevalence of advanced cervical cancer and limit the generalizability of results.

**Data handling and collection**: All patients underwent contrast-enhanced CT of the abdomen and pelvis on a multi-detector General Electric (GE) CT scanner according to departmental protocols. A radiology subspecialist and a senior resident reviewed the CT studies. Imaging findings related to the primary tumor, locoregional extent, lymphadenopathy, and metastasis were evaluated.

CT identified cervical tumors as poorly enhancing lesions within normally enhancing stroma or as non-specific cervical enlargement. Signs of parametrial invasion were noted through various imaging indicators. Hydronephrosis and ureteric involvement were assessed through CT findings. Sociodemographic and clinical data were extracted from TASH's digital recording system.

**Data analysis and quality assurance**: Collected data were entered into an Excel spreadsheet via Google Forms. Missing values, outliers, and inconsistencies were addressed. Cleaned data were analyzed using SPSS version 27, performing descriptive and reliability analyses. Weighted Cohen's Kappa was used to assess the agreement between clinical and imaging-based staging.

**Variables**: Dependent variables included FIGO-based clinical and CT staging at diagnosis, agreement between FIGO-based clinical and CT staging, and discrepancies between CT and MRI. Independent variables encompassed sociodemographic data, clinical information (HIV status, initial symptoms, time to diagnosis, histologic subtype), and imaging findings.

### Operational definition

Cervical Cancer: Malignant growth of cells in the cervix confirmed by histopathology.

Clinical Staging: Tumors staged according to FIGO guidelines.

Advanced Stage at Diagnosis: Participants diagnosed with stage III to IV disease.

Time Intervals: Duration from the onset of initial symptoms to the healthcare visit.

Ethical Consideration: Ethical approval was obtained from the Research and Ethics Committee of the Department of Radiology. Confidentiality was ensured by anonymizing patient information and restricting access to involved researchers.

## Results

**Sociodemographic and clinical characteristics**: A total of 115 participants were included, with a mean age of 53 ± 12 years; 54.8% were aged 41 to 60 years. The majority (57.4%) lived in rural areas, and nearly all (98.3%) had never undergone cervical cancer screening. Squamous cell carcinoma was the most common histological subtype (83.5%). Only 17.4% of patients were HIV positive. Initial symptoms varied, with vaginal bleeding (43.5%) and foul-smelling discharge (41.7%) being prevalent. Delay in seeking medical attention was noted, with 27% waiting 6–12 weeks, and only 5.2% presenting within 4 weeks (Table 2).

**Table 2:** Socio-demographic and clinical characteristics of study participants, Tikur Anbessa Specialized Hospital, Addis Ababa, Ethiopia September 01, 2022 - February 30, 2023 (N=115)

variables	N	(%)
**Age**		
**< 30 years**	1	0.9
**31 to 40 years**	21	18.3
**41 to 50 years**	32	27.8
**51 to 60 years**	31	27.0
**> 60 years**	30	26.1
**Place of Residence**		
**Urban**	49	42.6
**Rural**	66	57.4
**Distance from TASH, Addis Ababa in Km**		
**Less than in 100km diameter**	44	38.3
**More than 100km diameter**	71	61.7
**HIV Serostatus**		
**Positive**	20	17.4
**Negative**	95	82.6
**Initial clinical Symptom**		
**Vaginal bleeding**	50	43.5
**Foul smelling vaginal discharge**	13	11.3
**Pain with sexual intercourse**	4	3.5
**>1 symptoms**	48	41.7
**Histologic Subtype**		
**SCC**	96	83.5
**Adenocarcinoma**	14	12.2
**Others**	5	4.3
**Time interval before diagnosis**		
**≤ 1 month**	6	5.2
**1-3 months**	25	21.7
**3-6 months**	29	25.2
**6-12 months**	31	27.0
**>12months**	24	20.9

**Clinical staging and imaging results**: At initial examination, 53% of patients were classified with advanced clinical staging. CT imaging revealed advanced disease in 85 patients (73.9%), with only 22 (20.5%) in early stages eligible for surgery (Table 3). CT detected tumor sizes >6 cm in 34.8% of cases, with local invasion of bladder or rectum in 42.6%. Hydronephrosis was present in 40% of cases, and distant metastasis was identified in 13%. MRI demonstrated the primary tumor in all 34 cases.

**Table 3:** Clinical and CT based staging, Tikur Anbessa Specialized Hospital, Addis Ababa, Ethiopia September 01, 2022 - February 30, 2023 (N=115)

variables	N	(%)
CT based stage		
CT Stage IIA	11	9.6
CT Stage IIB	11	9.6
CT Stage IIIA	3	2.6
CT Stage HIB	16	13.9
CT Stage IIIC	11	9.6
CT Stage IVA	42	36.5
CT Stage IVB	13	11.3
Not determined	8	7.0
Clinical stage		
Stage IB	2	1.7
Stage IIA	17	14.8
Stage IIB	30	26.1
Stage IIIA	15	13.0
Stage IIIB	24	20.9
Stage IIIC	3	2.6
Stage IVA	19	16.5
Not determined	5	

**Staging discrepancies between clinical and imaging methods**: The study found that 60% of patients showed discrepancies in CT staging. MRI revealed similar discrepancies in 53% of cases (Table 4). Both modalities confirmed bladder/rectum invasion in 9 cases, with additional discrepancies in two cases where CT was uncertain.

**Table 4:** Staging comparison between Clinical Vs. imaging based staging, Tikur Anbessa Specialized Hospital, Addis Ababa, Ethiopia September 01, 2022 - February 30, 2023 (N=115)

variables	N	(%)
Clinical Vs. CT based staging		
CT over staged the disease	55	47.8
CT under staged the disease	14	12.2
The clinical and CT staging were similar	46	40.0
Total	115	100.0
Clinical Vs. MRI based local staging		
MRI over staged the local staging	16	13.9
MRI under staged the local staging compared	3	2.6
The clinical and MRI staging were similar	15	13.0
Total	34	29.6
Missing	81	70.4

**Agreement between clinical and imaging-based staging**: Reliability analysis indicated a poor agreement (weighted Cohen's kappa = 0.171, p = 0.016) between clinical and CT staging, while agreement with MRI staging was moderate (weighted Cohen's kappa = 0.418, p = 0.007) (Table 5).

**Table 5:** Agreement between Clinical Vs. imaging based staging, Tikur Anbessa Specialized Hospital, Addis Ababa, Ethiopia September 01, 2022 - February 30, 2023

Agreement between clinical Vs.	Weighted kappa value	95% CI	Significance
CT based staging (N-108)	0.171	0.032- 0.310	0.016
MRI based staging (N-34)	0.418	0.177- 0.658	0.007

## Discussion

This study aimed to evaluate the initial staging of newly diagnosed cervical cancer patients using clinical and imaging methods, highlighting discrepancies and agreement between these approaches. The average age of participants (53 ± 12 years) is consistent with previous studies, and vaginal bleeding was the most common presenting symptom.

Findings indicate that 53% of cases presented with advanced clinical staging, with CT-based staging revealing a higher rate of advanced disease at 85%. These results align with other studies reporting high prevalence rates of advanced cervical cancer in Ethiopia. Delayed diagnosis, low awareness, and rural residence were significant factors associated with late-stage disease.

The discrepancies observed between clinical and imaging methods underscore the limitations of clinical staging alone, particularly in assessing local and distant spread. The poor agreement between clinical and CT-based staging suggests that reliance on clinical evaluation can lead to under-staging. In contrast, the moderate agreement with MRI-based staging indicates its superior role in evaluating parametrial and lymph node involvement.

Given the high prevalence of advanced-stage cervical cancer, the study highlights the need for improved screening programs, early detection strategies, and enhanced imaging capabilities to better inform clinical decisions.

In conclusion, the study demonstrates that a significant proportion of cervical cancer patients present with advanced-stage disease. While CT staging often indicates a more advanced disease than clinical evaluation, MRI staging aligns more closely with clinical assessments. There is a clear need for improved early detection and staging methods to enhance patient management in cervical cancer.
